# Early Stage Melanoma of the Vermillion with Mucosal Melanoma in Situ: A Clinical Conundrum

**DOI:** 10.1007/s12105-023-01552-y

**Published:** 2023-05-19

**Authors:** Sasha J. Betz, Jonathan J. Jelmini, Roderick Y. Kim

**Affiliations:** 1 Oral and Maxillofacial Surgery, Naval Medical Center San Diego, 34800 Bob Wilson Drive, San Diego, CA 92134 USA; 2 Division of Maxillofacial Oncology and Reconstructive Surgery, Department of Oral and Maxillofacial Surgery, John Peter Smith Hospital, 1500 South Main Street, Fort Worth, TX 76104 USA

**Keywords:** Melanoma staging, Mucosal melanoma, Vermillion melanoma, Mucosal melanoma in situ

## Abstract

**Background:**

Melanoma is a predominantly cutaneous malignancy associated with sun exposure. Mucosal melanoma is rare and carries a distinct pathogenesis from cutaneous tumors. The vermillion of the lip is a unique site that divides cutaneous from mucosal tissues. Tumors arising on the dry aspect are classified as cutaneous and those of the wet aspect are mucosal. The distinction is importation in tumor staging as all mucosal melanomas are classified as T3-T4b under the current 8th edition American Joint Committee of Cancer (AJCC) guidelines.

**Methods:**

We describe a case of early stage melanoma of the vermillion with associated mucosal melanoma in situ. We discuss nuances of management at this site and the distinctions between cutaneous versus mucosal melanomas with a review of the literature.

**Results:**

Our patient was treated surgically with 2-3 cm margins. Residual melanoma in situ was present at the mucosal margin on final pathology which required a second surgery for margin revision. The case was discussed at tumor board with recommendation for no further treatment.

**Conclusions:**

The nuances between the vermillion and mucosal lip must be understood for appropriate staging and treatment of melanomas. The paucity of literature on melanomas affecting this site render management decisions challenging. Multidisciplinary discussion is essential for guiding care.

## Introduction

Melanoma is a predominantly cutaneous malignancy that affects approximately 100,000 United States citizens annually [1]. These malignancies arise from melanocytes, pluripotent neural crest cells that proliferate and migrate via various developmental pathways to colonize the ectoderm and endoderm [2]. The pathogenesis of melanoma varies according to subtype with sun exposure being the most significant etiologic factor [3].

Tumors arising at mucosal sites are rare and account for only 1–4% of all melanomas [2]. Mucosal melanoma (MM) occurs in the genitalia, oral and nasal cavities, and conjunctiva [3]. The etiology of MM is unknown as the sites affected are protected from sun and there is limited evidence of chemical or viral etiologies [3].

The lip is a unique anatomic site that can be divided by the wet-dry line into the mucosal lip and vermillion. The distinction is important in tumor staging; cancers of the dry vermillion are classified with cutaneous malignancies and those of the mucosal lip are classified as oral cavity malignancies [4]. Due to the aggressive nature, even of small tumors, the AJCC TNM staging system for MM begins at T3. Mucosal melanoma in situ (MIS) is excluded from the staging criteria due to its rare occurrence [4].

We report a case of early stage melanoma involving both the mucosal and vermillion surfaces of the lower lip. We discuss the nuances of tumor staging at this site and challenges of surgical management.

## Case Report

A 48-year-old male was referred to our clinic from an outside provider for a pigmented lesion of the lower lip. The patient reported first noticing it five years prior with progressive enlargement over time. He spent time outdoors as a hunter and reported sunscreen use but did not consciously apply sun protection to his lips. He denied self or family history of skin cancers. His medical and surgical history were non-contributary. He chewed tobacco and was a heavy consumer of alcohol. His review of systems was unremarkable.

**Fig. 1:**
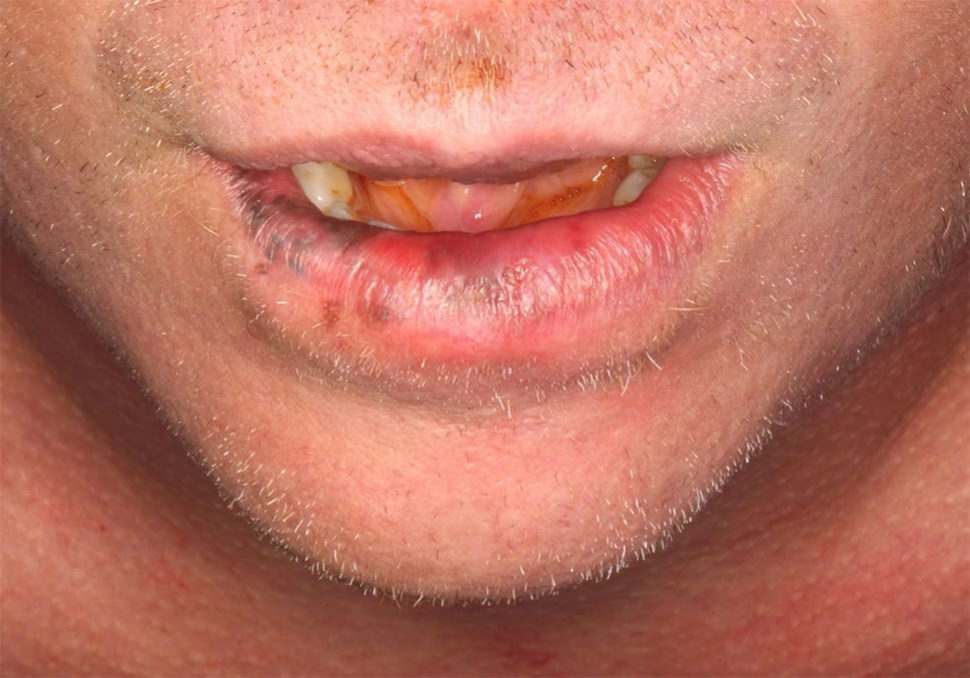
Heterogeneous pigmentation primarily affecting the vermillion of the lower lip, extending to the white roll

**Fig. 2:**
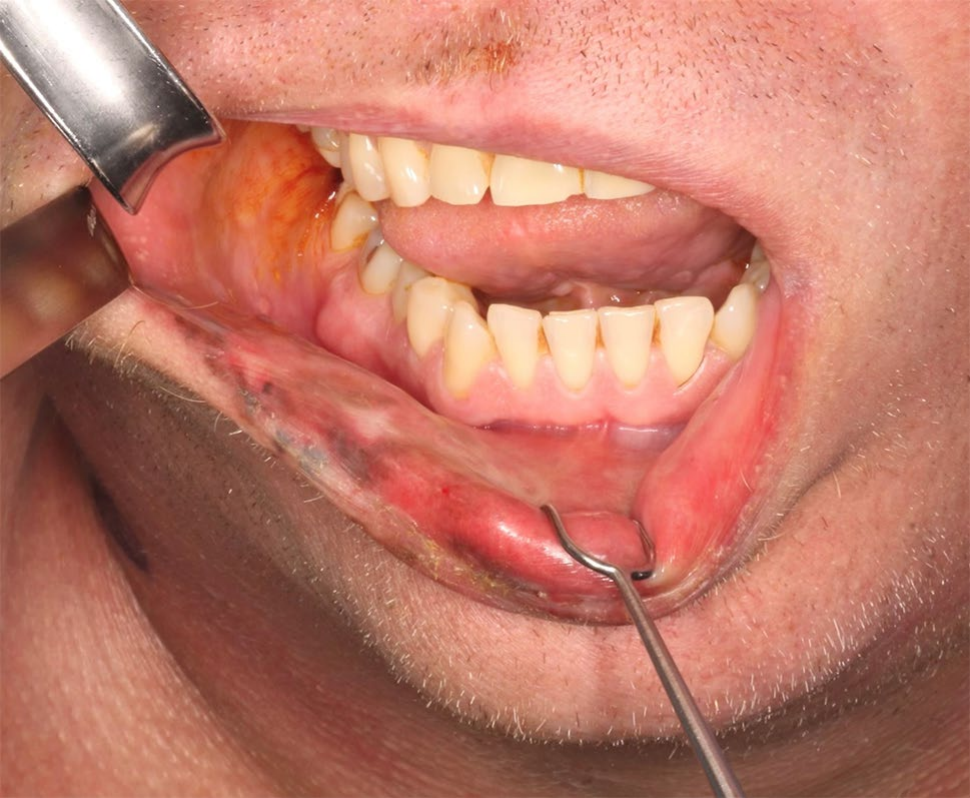
Heterogeneous pigmentation primarily affecting the vermillion of the lower lip, extending to the mucosa

On clinical examination, he was Fitzpatrick I with reddish hair and blue-grey eyes. A heterogenous black to brown macular lesion involved approximately three quarters of the lower lip (Figs. 1 and 2). It spanned from the right labial commissure past midline and demonstrated ill-defined, irregular borders. It involved the white roll, dry vermillion, and mucosal lip. No raised masses or nodules were palpable. No cervical lymphadenopathy was appreciated. His oral cavity and head and neck examination was otherwise unremarkable.

**Fig. 3:**
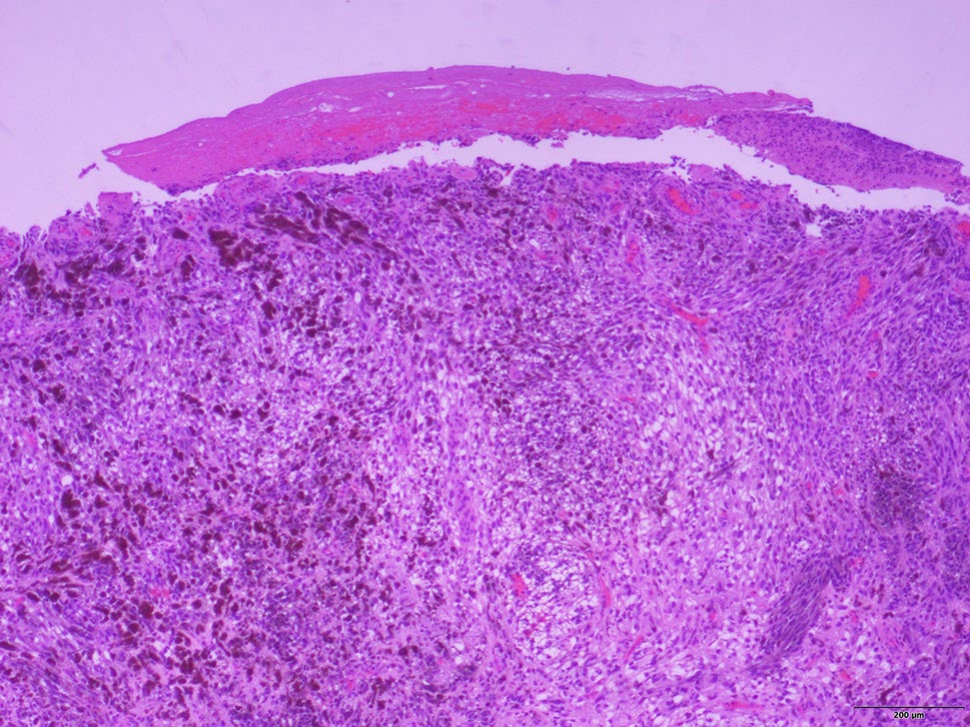
Surface ulceration overlying neoplastic cells in a haphazard arrangement of nests and fascicles (hematoxylin and eosin, 4 × magnification). *Photomicrograph credit: Dr. Lisa Cheng*

**Fig. 4:**
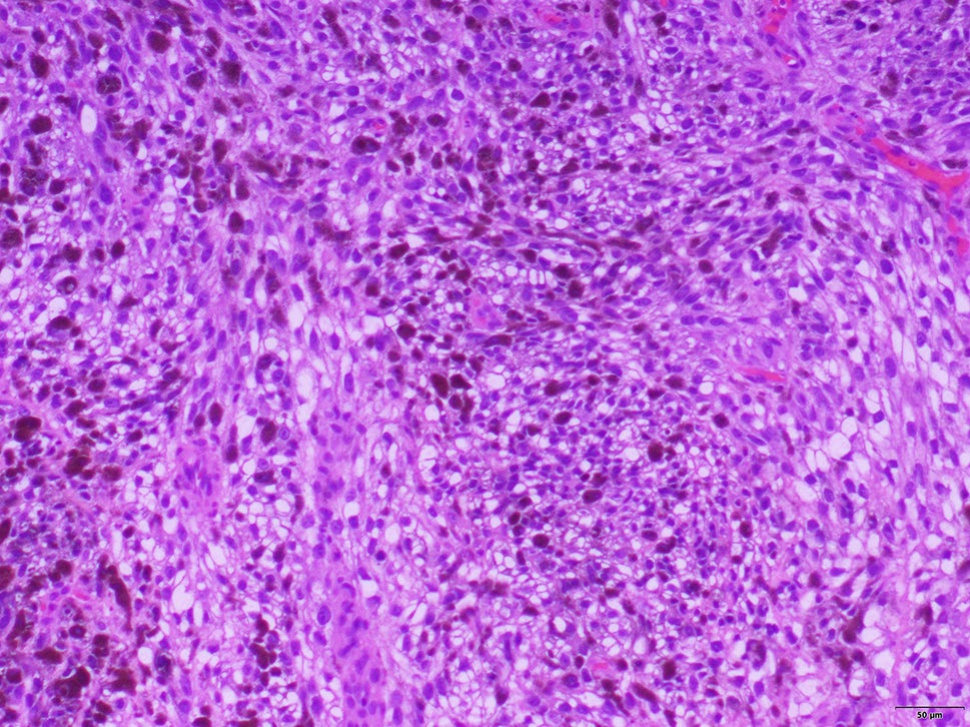
Round to spindled tumor cells with clear to amphophilic cytoplasm. A subset show irregular nuclear contours with prominent nucleoli. Individual cells demonstrate heavy melanin production (hematoxylin and eosin, 10 × magnification). *Photomicrograph credit: Dr. Lisa Cheng*

**Fig. 5:**
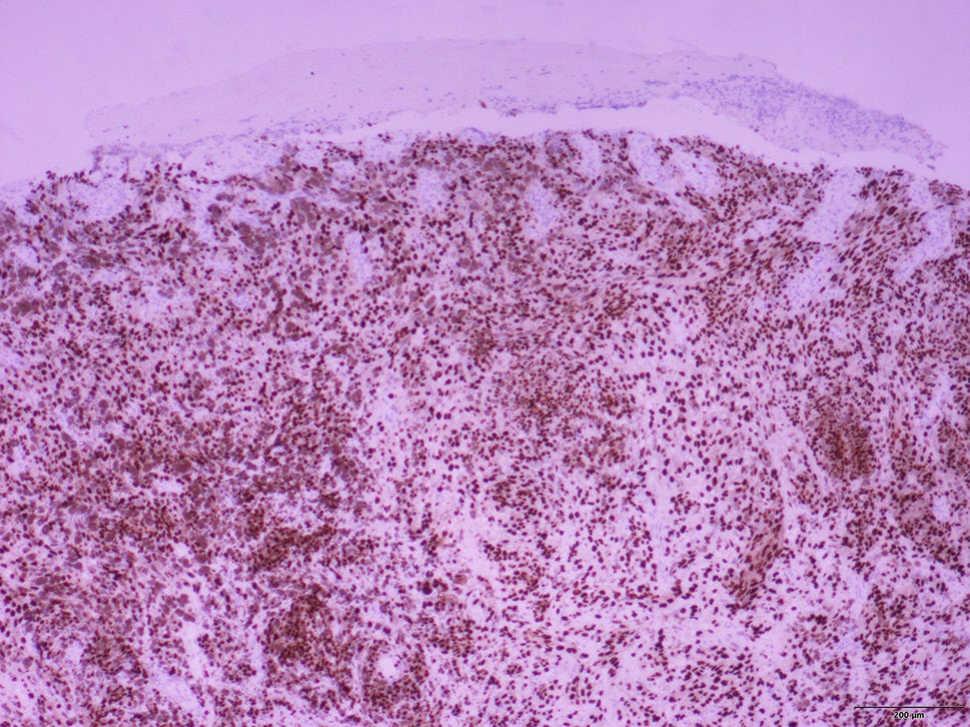
SOX10 highlights the nuclei of invasive malignant melanocytic cells (SOX10 immunohistochemisty, 10 × magnification). *Photomicrograph credit: Dr. Lisa Cheng*

An incisional biopsy was performed by the referring provider. The specimen was surfaced by ulcerated stratified squamous epithelium overlying a neoplasm comprising round to elongated cells, some with heavy melanin production (Fig. 3). Irregular nuclear contours with prominent nucleoli were present (Fig. 4). The adjacent connective tissues demonstrated actinic elastosis suggestive of solar damage. Immunohistochemical staining for SOX10 was positive indicating neural crest and melanocytic origin (Fig. 5). A diagnosis of malignant melanoma with ulceration was rendered.

The patient underwent positron emission tomography (PET) for staging purposes. The primary lesion was not identifiable on the scan and there was no evidence of metastasis. A repeat biopsy was performed by our institution to confirm the diagnosis and stage the tumor. The biopsy was staged as a cutaneous melanoma (CM); pT1a with 0.5 mm tumor thickness.

**Fig. 6:**
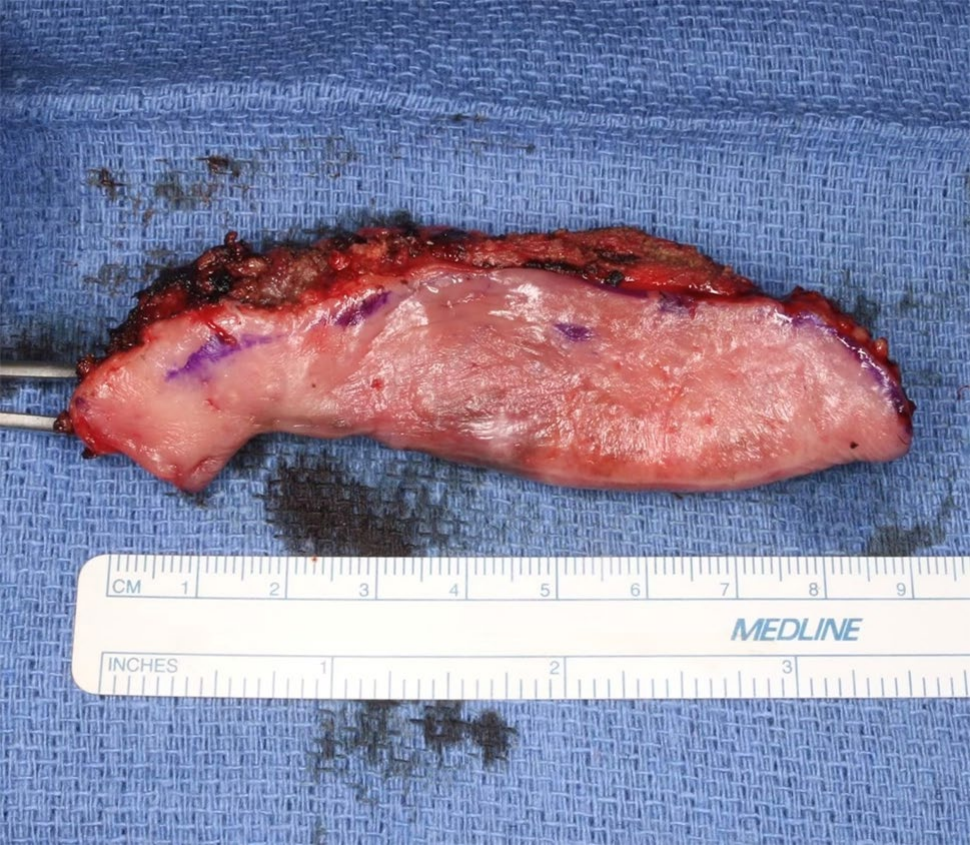
Mucosal aspect of the resection specimen with 2-3 cm margins

The patient was taken to the operating room for wide local excision with 2 to 3 cm margins (Fig. 6) and selective neck dissection of levels 1–3, excluding 2b. Intraoperative pathology consultation with frozen sections showed negative margins. The site was immediately reconstructed with a radial forearm free flap.

**Fig. 7:**
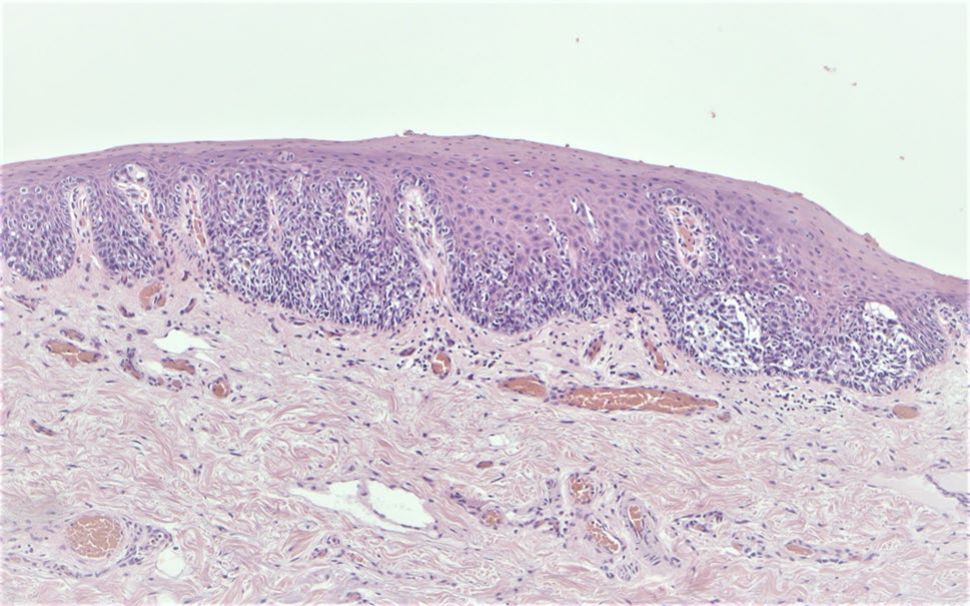
Junctional nests of neoplastic melanocytes in the mucosal melanoma in situ aspect of the specimen (hematoxylin and eosin, 10 × magnification). *Photomicrograph credit: Dr. Katherine Tumminello*

The final pathology showed MIS with wide involvement of the mucosal margin (Fig. 7). There was no evidence of spread to regional lymph nodes. It was confirmed with the pathologist that since only MIS was appreciated on definitive resection, the staging was deferred to that of the biopsies; pT1bN0 due to ulceration on the first biopsy.

The patient was returned to the operating room for margin revision. No lesional tissue was clinically apparent, so additional margins were taken along the entirety of the mucosal aspect of the free flap. The pathology showed residual MIS which was superseded by a negative new margin. The case was presented at multidisciplinary tumor board with recommendations for no further treatment.

## Discussion

Mucosal melanoma is a rare but highly aggressive tumor with distinct pathogenesis, clinical course, and molecular profile from that of CM [6]. Whereas CM is caused by sun exposure, MM occurs in sun-protected sites and no etiologic factor is proven to be causative [2, 5]. Fair skin, presence of multiple or dysplastic nevi, and family history of melanoma are risk factors for CM [6]. In contrast, no clear risk factors are defined in MM and an increased incidence in Japanese and African populations is reported [5]. The most common intraoral sites affected are the hard palate, maxillary alveolus, and mandibular alveolus [7]. The 5-year survival rate for MM of the head and neck is 20–40% overall, and that of oral MM is particularly poor at 15% [5]. The 5-year survival of CM is comparatively favorable at 80.8% [6].

The staging of MM differs considerably from that of CM by the 8th edition AJCC guidelines. In CM, the T-categories range from pT0-pT4 and are based on Breslow thickness, the measurement from the granular layer of the epidermis to the deepest point of invasion, as well as ulceration [10]. Histologic features such as mitotic rate, lymphovascular invasion, and neurotropism are reported but not weighed in the T-category [10]. In contrast, the T-category for MM begins at pT3 which describes tumors limited to mucosa and the immediate underlying tissues [4]. The pT4a category describes tumors involving deeper tissues with pT4b representing very advanced disease [4].

Our patient was staged as a CM, pT1bN0. This was based on the biopsies of the clinically most affected areas which comprised the dry vermillion. Adverse features included ulceration and 1 mitotic figure per mm^2^; however, only MIS was identified in the resection specimen including the mucosal lip. The development of melanoma on a sun-exposed area in a Fitzpatrick I patient with histologic evidence of actinic damage further supported a cutaneous classification. Per National Comprehensive Cancer Network guidelines (NCCN), the tumor was clinical stage IB and the indicated treatment was wide local excision [8].

Wide local excision with clear margins is the mainstay treatment for both CM and MM [8, 11]. In MM, the survival advantage of clear margins is apparent, with one study showing positive margins associated with up to 21-fold increased risk of death due to disease [12]. The extent of surgical margins for CM varies according to tumor thickness. The recommended margins for MIS is 0.5–1.0 cm, 1.0 cm for tumors less than 2 mm, and 2.0 cm for lesions thicker than 2 mm [6]. The recommended margins for MM is not well defined, but some have advocated for at least 1.5 cm [12].

The surgical team elected for wider margins at 2.0–3.0 cm to account for the mucosal involvement. Intraoperative pathology consultation was attempted to determine margin status; however, there was disagreement between the frozen section and permanent section results. This was unfortunate but not unexpected, as frozen sectioning diminishes the histologic quality and makes the pathologic interpretation of subtle lesions, such as MIS, exceedingly challenging [10]. The decreased histologic quality results in low concordance between frozen section and permanent margin interpretation for melanocytic lesions [6, 10]. Despite wider margins and intraoperative pathology consultation, the final specimen mucosal margin was positive for MIS and necessitated margin revision.

Per NCCN guidelines, sentinel lymph node biopsy may be recommended for pT1b tumors after discussion with the patient [9]. The guidelines for lymph node biopsy and management of the neck are less defined in MM, but it is noted that depth of invasion, clinical judgement, reliability of follow-up, and clinical suspicion should be utilized to guide decision-making [11]. In our case, elective neck dissection of levels 1–3, excluding 2B, was opted for at time of resection. No nodal involvement was identified on final pathology.

In CM, primary site radiotherapy (RT) is reserved for medically inoperable patients or in instances where surgical morbidity of resection is prohibitive [8]. In MM, it is recommended that primary site RT be considered, especially in sites of suspected subclinical spread; however, studies have shown a benefit to local control but not improved overall 5-year survival [11, 12].

Current guidelines do not recommend routine molecular testing or systemic therapy for neoadjuvant treatment of early stage, clinical stage I–II, resectable CM outside of clinical trials [8, 13]. For patients with resected stage III or higher disease, systemic therapy is recommended based on the molecular profile of the tumor [8, 13]. *BRAF* testing is recommended for stage III patients in whom *BRAF*-directed therapy may be an option. For patients with stage IV disease or recurrent disease, *BRAF* specific testing or Next Generation Sequencing (NGS) for broader genomic profiling is recommended [8]. The molecular profile of MM is more variable that that for CM, but mutation of *BRAF* is only seen in 3% of cases [7]. Evidence for systemic therapy in MM is lacking, and adjuvant therapies have failed to prove prolonged overall survival [11, 12]. Nonetheless, the NCCN guidelines for MM of all stages recommend adjuvant systemic therapy but admits the evidence for it is less than that of cutaneous melanoma [11]. The specific treatments recommended vary on extent of disease and molecular profile [12].

There is a paucity of literature evaluating melanoma of the lip overall with only seven cases specifying the vermillion as the primary site [14]. Additional cases reported may have affected the vermillion primarily but were classified by their authors as mucosal [15–18]. A study of 48 patients with lip melanoma staged all cases as MM without distinction between vermillion versus mucosal involvement [15]. Tumors were treated with wide resection with 1.5–2.0 cm margins and a subset received adjuvant chemotherapy. Neck dissection was performed in stage IV patients. Interestingly, most patients had a long history of lip melanin pigmentation prior to diagnosis like our patient [15]. Macular melanomas had a 5-year survival rate of 100% as compared to nodular tumors at 29%. There was no 5-year survival benefit to stage III patients who received chemotherapy compared to those who were treated with surgery only [15]. Overall, patients had improved 5-year survival rate compared to those reported for all-comer mucosal melanomas at 56.1% [15]. In our case, since only MIS was identified on the final resection, no additional treatment was recommended. The lack of survival benefit of chemotherapy to stage III patients supports surgery as the primary treatment modality.

The tumor included in this study was early stage with a mucosal MIS component. Similarly, a recent study of 170 patients with oral MM describes 22 patients with superficial invasion or MIS (Clarks levels I–II) [19]. Most had macular tumor morphology (18/22) but four had nodular morphology with a tumor size ≤ 1 cm. All patients were staged as pT3N0M0 per 8th edition AJCC criteria. All were treated with resection with at least 1 cm margins, and the nodular MM received prophylactic neck dissections. Nineteen patients received post-operative therapy: chemotherapy in 16 and radiation therapy in three. The 5-year overall survival (OS) in this group was 90%. In those that received adjuvant therapy, the OS was 100% [19]. The authors proposed de-escalation of staging for these early presentations of MM in the next edition of AJCC staging criteria [19].

## Conclusion

The lip is a unique site that divides mucosa from skin. When tumors involve both the mucosa and vermillion, the implications for staging and treatment must be understood. This is especially true in melanoma due to the vast differences in staging and management between CM and MM. The paucity of literature on melanomas affecting this site and the nuances of staging render management decisions challenging. Multidisciplinary discussion is essential for accurate staging and determining best treatment.

## Data Availability

Not applicable.
